# Persistent primitive trigeminal artery in stroke: A review and clinical insights

**DOI:** 10.3934/Neuroscience.2025024

**Published:** 2025-09-28

**Authors:** Jad El Choueiri, Francesca Pellicanò, Lorenzo De Rossi, Victor Gabriel El-Hajj, Lorenzo Pellegrini, Leonardo Di Cosmo, Anthony Abi Chakra, Nakul Gokul Varma, Francesco Laurelli, Maria Pia Tropeano, Zefferino Rossini, Federico Pessina, Gabriele Capo

**Affiliations:** 1 School of Medicine, Humanitas University, Via Rita Levi Montalcini 4, 20072, Pieve Emanuele-Milan, Italy; 2 Department of Clinical Neuroscience, Karolinska Institutet, Stockholm, Sweden; 3 Faculty of Agricultural and Food Sciences, American University of Beirut, Beirut, Lebanon; 4 Department of Neurosurgery, IRCCS Humanitas Research Hospital, via Manzoni 56, 20089 Rozzano, Milan, Italy; 5 Department of Biomedical Sciences, Humanitas University, Pieve Emanuele, Milan, Italy

**Keywords:** persistent primitive trigeminal artery, stroke, vascular anomalies, neuroimaging, hemodynamics, anatomy

## Abstract

The persistent primitive trigeminal artery is the most common remnant of the embryonic carotid–basilar connection. While its anatomy is well documented, its clinical significance in strokes remains debated. Some studies suggest it predisposes ischemia through altered hemodynamics, whereas others report a protective role via collateral circulation. This review summarizes the current knowledge on the anatomy, imaging, and clinical implications of the persistent primitive trigeminal artery in strokes. A literature search of PubMed, Scopus, and Embase up to March 2025 identified relevant case reports, imaging studies, and clinical series. The artery typically arises from the cavernous segment of the internal carotid artery, courses alongside the trigeminal nerve, and terminates in the posterior circulation. Classification systems describe variations in its termination, course, and vascular supply. Magnetic resonance and computed tomography angiography are key diagnostic tools, each with their advantages and limitations. Evidence shows two opposing roles: either as a conduit for steal phenomena, turbulent flow, and thromboembolism or as a collateral channel that preserves cerebral perfusion during carotid or vertebrobasilar occlusion. Understanding this vessel's anatomy and potential hemodynamic effects are critical for accurate diagnoses, risk assessments, and treatment planning. Its dual nature highlights the importance of individualized evaluations and further research to clarify its role in strokes.

## 1. Introduction

The trigeminal artery forms during embryological development as one of the 4 transient anastomotic channels (trigeminal, otic, hypoglossal, and proatlantal intersegmental arteries) between the carotid and the paired longitudinal neural arteries (PLNAs), which later come together to form the basilar artery [1]. Padget's embryological study [2] described the trigeminal artery as initially being a branch from the dorsal aorta, connecting to the longitudinal neural arterial plexus. During the development of the basilar artery (BA) system, the PLNAs are mainly fed at the cephalic ends by the trigeminal arteries, as well as the primitive hypoglossal and proatlantal intersegmental arteries. Thus, the trigeminal arteries have the essential function of supplying blood to the hindbrain of the embryo in the absence of posterior communicating (PCom) and vertebral arteries, which are yet to form [3],[4].

Under normal circumstances, the trigeminal artery regresses by approximately the sixth week of embryonic development [5] as the vertebrobasilar system matures; however, in some cases, it remains into adulthood and is hence referred to as either the persistent primitive trigeminal artery (PPTA) or the persistent trigeminal artery (PTA). Therefore, the PTA is an anastomotic vessel between the internal carotid artery (ICA) and the BA [6] that typically originates from the cavernous segment of the ICA [7], although more proximal origins from the petrous segment of the ICA have been reported [1],[8]. It is the most common type of the persistent carotid-basilar anastomotic vessels [9], accounting for 80%–85% of these remnants, and has a reported prevalence of 0.061%–0.6% in the population [1].

As a carotid-basilar anastomosis, the PTA can reportedly have a significant impact on the cerebrovascular hemodynamics [10]. In addition, its presence in adults may have important clinical implications, including PTA saccular aneurysms that occur in 14%–32% of PTAs [1],[11] and trigeminal-cavernous sinus fistulas, which result from either a traumatic or an aneurysmal rupture of the PTA. Moreover, the PTA may play a role in Moyamoya disease [1], and can be associated with trigeminal neuralgia as a result of compression of the trigeminal nerve [12] in their parallel course. Additionally, the disruption of the cerebrovascular hemodynamics associated with a PTA in some specific variants has been reported to cause developmental issues such as hypoplasia or agenesis of the PCom [3]. Therefore, it is essential to understand the anatomy and effect of this remnant vessel in order to recognize the clinical implications, make an accurate diagnosis, and provide a timely management of the associated conditions.

While the implication of the PTA in several conditions has been fairly described [1], the literature surrounding its involvement as a protective or risk factor in the pathophysiology of strokes is still contradictory, and has yet to be comprehensively summarized. It has been documented that the PTA can contribute to the occurrence of an ischemic stroke; however, other cases have shown an opposite association. Hence, this review primarily aims to discuss and provide a synthesis regarding the role played by the PTA in the context of ischemic strokes primarily, with hemorrhagic events being discussed only when attributable to the PTA pathology. Understanding the role of the PTA in the pathophysiology of ischemic strokes may aid in both the prognostication and risk management of affected patients.

## 2. Materials and methods

A comprehensive literature search was conducted using the PubMed, Scopus, and Embase databases to identify studies linking PTA to cerebrovascular events, particularly stroke. The keywords used included but were not limited to “PTA”, “PPTA”, “Stroke”, “ischemic stroke”, and “cerebrovascular events”. This review was designed as a narrative review rather than a systematic review; therefore, the Preferred Reporting Items for Systematic reviews and Meta-Analyses (PRISMA) guidelines were not formally applied, and no risk of bias assessment or Grading of Recommendations Assessment, Development and Evaluation (GRADE) evaluation was performed. The search was limited to articles published up to March 2025 which discussed the relation between the presence of the PTA and the occurrence of strokes. The inclusion criterium was peer-reviewed human studies that reported on the PTA and strokes, including case reports, case series, and retrospective studies. Studies were included regardless of the language if the full text was available. The exclusion criteria were as follows: animal or cadaveric studies; technical notes without clinical data; abstracts without a published article; and duplicate publications. The articles were screened by title and abstract by two independent authors, and the full texts of the articles were reviewed to extract the relevant data about the diagnostic methods used and the suggested relation between the PTA and strokes. For each included study, we extracted the following data when available: first author, year of publication, study type, number of cases, PTA type, stroke subtype, proposed stroke mechanism, and imaging modality. The primary outcome was an ischemic stroke. The secondary outcomes included a hemorrhagic stroke (SAH) only when it could directly be linked to the PPTA pathology. Unruptured aneurysms were not counted as stroke events. Given the heterogeneity across the reports, the data were summarized in a narrative synthesis. Any discrepancies between the data collected by the two authors were either resolved by consensus or by consultation with the senior author.

## 3. Results and discussion

### 3.1. Anatomy and classification

The artery was first described by Richard Quain in 1844 [13]; since then, its anatomical course, variations, and clinical significance have been increasingly elucidated.

The PTA most commonly emerges from the cavernous segment of the ICA [14], travels parallel to the trigeminal nerve within Meckel's cave, hence the name, and extends towards the posterior circulation [14],[15], ultimately terminating in the BA. This course is variable, with differences in its origin, termination, and anatomical relationships to adjacent structures (such as the abducens nerve) being documented in the literature [8]. This led to the development of multiple classification systems aimed at categorizing the PTA (Table 1).

The most prevalent of the classification models includes Saltzman's classification, which was first proposed in 1959 and details subtypes based on the termination of the PTA into the BA [16]. A Saltzmann type I PTA joins the BA between the anterior inferior cerebellar artery (AICA) and the superior cerebellar artery (SCA). It can be associated with hypoplasia of the PCom, the vertebral artery (VA) on the same side, and the proximal BA.

A Saltzman type II PTA inserts proximally to the SCA and supplies it. The BA, the VAs, and the PCom are usually well formed. A Saltzmann type III PTA terminates in one of the 3 cerebellar arteries without any anastomoses with the BA. Specifically, type IIIa terminates in the SCA, type IIIb, which is the most common variant, terminates in the AICA, and type IIIc terminates in the posterior inferior cerebellar artery (PICA) [1],[10]. One persistent trigeminal artery variant (PTAV) is a rare variant in which the cerebellar arteries directly originate from the ICA without an intervening connection of the BA [9]. Their relationship to strokes can be multifactorial through mechanisms such as hemodynamic steal, a paradoxical embolism from the carotid to the posterior fossa, and potentially decreased hemodynamic stability.

A subsequent classification was proposed by Salas et al. in 1998, where the team classified the PTA based on its course [15], thereby distinguishing between a medial or sphenoidal type and a lateral or petrosal type. In a subsequent study, Weon et al. (2011) [17] proposed a further more nuanced classification scheme, thereby building on the Saltzman's system and using findings from magnetic resonance angiography (MRA) findings to help distinguish the PTA types by the vascular supply patterns.

**Table 1.** Classification of PTA based on Saltzman (1959), Salas et al. (1998) and Weon (2011) [15]–[17].

**Classification system**	**Type**	**Anatomical description**
**Saltzman (1959) [16]**	Type I (fetal type)	PTA connects ICA to BA between the SCA and the AICA; proximal BA, Pcom, and VA may be hypoplastic
	Type II	PTA connects ICA to BA proximally to the SCA and supplies it; distal BA is intact
	Type III	PTA terminates as a cerebellar artery (SCA, AICA, or PICA)
**Salas et al. (1998) [15]**	Medial (sphenoidal) type	PTA courses through the sella turcica
	Lateral (petrosal) type	PTA courses lateral to the dorsum sellae
**Weon (2011) [17]**	Type 1	PTA supplies BA, PCA, and SCA Corresponds to Saltzman type I
	Type 2	PTA supplies SCA, PCA is supplied via the PCom Corresponds to Saltzman type II
	Type 3	PTA supplies contralateral PCA, ipsilateral PCA is supplied via the PCom
	Type 4	PTA supplies ipsilateral PCA, contralateral PCA is supplied via the PCom
	Type 5a, b, c	Trigemino-cerebellar terminations (Equivalent to Saltzman Type III)

### 3.2. Imaging and diagnosis

The presence of a PTA can be detected using MRA and computed tomography angiography (CTA). Both techniques can be highly effective in the individuation of this embryonic vascular remnant thanks to their efficacy in visualizing the vascular anatomy.

MRA is extremely useful given its non-invasive nature and high-resolution capabilities. With its three-dimensional (3D) time-of-flight (TOF) and contrast-enhanced techniques, it can clearly delineate the origin, course, and termination of the PTA and its variants, as well as any associated vascular anomalies [18],[19].

CTA provides detailed images of the vascular structures, thus emphasizing its importance in identifying vascular pathologies and planning neurovascular interventions [20].

Even though their efficacy is backed by several studies, both of these modalities still bear some limitations. In particular, 3D TOF MRA has been reported to have a lower spatial resolution compared to CTA, which can limit the visualization, especially in specific variants of PTA where small vascular structures and complex anatomical relationships ought to be observed. Additionally, it is slower than CTA, thus limiting its use in emergency settings. Moreover, 3D TOF MRA is susceptible to flow-related artifacts, which can obscure or even mimic vascular anomalies, thus leading to an increase in the number of false positives or negatives [21],[22].

On the other hand, CTA involves the exposure of the patient to ionizing radiation, which can be contraindicated in young subjects or those that require extensive follow-up routines. Additionally, CTA requires the use of iodinated contrast agents, which can pose risks of allergic reactions or nephrotoxicity in patients with a pre-existing renal impairment [23],[24]. While its quality may be superior to MRA, CTA provides static images which potentially limit the assessment of certain vascular pathologies or anomalies, including PTAs [25]. This could consequently limit the accuracy of the PTA classification, as the hemodynamic state of the vessel itself and its terminal end are key points of information for this classification.

Given the known association between the PTA and cerebrovascular pathologies such as aneurysms, arteriovenous malformations, and trigeminal neuralgia, its detection with imaging is fundamental to avoid a misdiagnosis and to guide an appropriate treatment [7],[26]–[29].

### 3.3. PTA as a risk factor of stroke

A review of the literature examining the link between the PTA and strokes reveals a complex interplay between its role as a contributing and protective factor. While the PTA itself is an embryonic remnant and not inherently pathological, certain configurations may predispose to or protect from strokes by altering hemodynamics.

The presence of a PTA may contribute to a stroke through several mechanisms, including steal phenomena and thrombosis in the anterior circulation. In general, the steal phenomenon is a pathophysiological process where blood flow is redirected from one vascular territory to another, which causes ischemia in the deprived area. In particular, it is typically due to the presence of a low-resistance vascular bed [30]. Focusing on the PTA, this artery connects the cavernous part of the ICA with the posterior circulation, thus altering the hemodynamics by preferentially shunting blood through the PTA, which results in a reduced perfusion to the anterior circulation and possibly a predisposition to ischemic events [31]. Additionally, the presence of a PTA may generate turbulent flow and predispose the formation of thrombi, which can then travel through the PTA and obstruct vessels in the anterior circulation, in turn, causing an ischemic stroke [31].

The following table summarizes the reports that explored the relationship between a PTA and strokes, which utilized various methodologies to establish its role as a causative factor (Table 2).

**Table 2.** Table summarizing studies exploring PTA as a causative factor for stroke.

Article	Type	Key Findings	Imaging Used	Conclusion
Caesar et al. [32]	Case report	Right Parietal lobe infarction after Left vertebral artery rupture and embolism using the PTA as a conduit	DWI, 3-D TOF MRA,	PTA as a stroke risk factor/unique case (associated to VA rupture)
Yin et al. [33]	Case report	Multiple-foci stroke and central retinal artery thrombosis (1) and pontine infarction, aneurysm, and unilateral hypoplasia of the vertebral artery (2)	cranial MRI, MRA, and fundus photography	PTA as a stroke risk factor
Iancu et al. [34]	Case report	Occlusive carotid dissection with extensive thrombosis within a persistent trigeminal artery	MRI, 3-D TOF MRA	PTA as a stroke risk factor
Ito et al. [35]	Case report	Superior cerebellar artery dissection leading to SAH in which the PPTA may have influenced the formation of SCA dissection	CT, Digital subtraction angiography	PTA as a stroke risk factor
Schwartz et al. [36]	Case report	left middle cerebral artery and posterior cerebral artery territories	MRA	PTA as a stroke risk factor
Ferreira et al. [31]	Case report	PTA probably associated to vertebrobasilar insufficiency	CT angiography	PTA responsible for ischemic events through steal phenomena or thrombosis in anterior circulation
Park et al. [37]	Case report	PTA stenosis as the primary cause of vertebrobasilar insufficiency in the absence of relevant ICA stenosis	MRA	PTA as a stroke risk factor
Yamamoto et al. [38]	Case report	Persistent PTA associated cerebral aneurysm rupture causing subarachnoid hemorrhage	CT, 3D-CTA	PTA as a stroke risk factor

### 3.4. PTA as a protective factor in stroke?

In contrast, since the PTA connects the cavernous segment of the ICA to the BA, it has been suggested to serve as a collateral pathway in cases of ICA occlusions. Therefore, by shunting blood from the posterior to the anterior circulation, the PTA may also play a protective role, thereby potentially preserving cerebral perfusion, limiting the extent of infarction, and reducing the severity of ischemic events (Table 3). For instance, Xu et al. reported a case where the PTA supplied the left middle cerebral artery and bilateral anterior cerebral arteries, which only resulted in minor ischemic events despite the ICA occlusion [27]. Similarly, Engelhardt et al. described a case where the PTA limited the extent of infarction following a traumatic ICA dissection [39]. The team elaborated on their findings, thereby stating that the higher the number of collaterals, the lesser the extent of the infarct.

However, the compensatory mechanism provided by the PTA is not consistently observed, as it might depend on the efficiency of the collateral circulation and the hemodynamic stability of the patient. In some cases, the PTA has been shown to not offer sufficient compensation, especially in cases with concomitant vascular anomalies or if the vessel itself is compromised [40].

**Table 3.** Table summarizing studies exploring PTA as a protective factor against stroke.

Article	Type	Key Findings	Imaging Used	Conclusion
Xu et al. [27]	Case report	PTA + ICA occlusion	DWI, Digital subtraction angiography, CT Perfusion	PTA as a protective mechanism
Nunes et al. [41]	Case report	Post Vertebral Artery Dissection Medullary stroke	DWI, Digital subtraction angiography	PTA as a protective mechanism
Ito et al. [42]	Follow-up case report	Collateral flow provided by PTA to pons able to avoid major damages caused by basilar artery occlusion	MR, cerebral angiography	PTA as a protective mechanism
Engelhardt et al. [39]	Case report	Limited infarction by the PTA following traumatic dissection of ICA	DWI, Digital subtraction angiography	PTA as a protective mechanism

## 4. Conclusions

The findings of this review underscore the importance of detailed imaging and clinical correlations to understand the hemodynamic implications of a PTA. Recognizing the role of the PTA in the context of cerebrovascular diseases is crucial to appropriately treat and therefore mitigate stroke risk.

Thus, a PTA remains a double-edged sword in the context of cerebrovascular health, with a seemingly significant interpatient variability. While PTAs have been identified as a potential risk factor for ischemic strokes in certain cases, their anastomotic nature has also been reported to confer protective effects against strokes in some patients. With respect to hemorrhagic strokes, the causative relationship between a PPTA and aneurysm formation remains debated, and the current evidence is insufficient to establish causality. Further studies are warranted before firm conclusions can be drawn. Large, prospective, and longitudinal studies of patients with and without PTAs are required to determine the exact clinical significance of this vessel on the pathophysiology of strokes.

## Use of AI tools declaration

The authors declare that GPT4 was used for rephrasing and clarification. The authors have rechecked all information and take full responsibility for the content of the manuscript.
